# Thermal acclimation of photosynthetic activity and RuBisCO content in two hybrid poplar clones

**DOI:** 10.1371/journal.pone.0206021

**Published:** 2019-02-11

**Authors:** Lahcen Benomar, Mohamed Taha Moutaoufik, Raed Elferjani, Nathalie Isabel, Annie DesRochers, Ahmed El Guellab, Rim Khlifa, Lala Amina Idrissi Hassania

**Affiliations:** 1 Faculté de foresterie, de géographie et de géomatique, Université Laval, Québec, QC, Canada; 2 Department of Biochemistry, University of Regina, Regina, SK, Canada; 3 Saskatoon Research and Development Centre, Agriculture and Agri-Food Canada, Saskatoon, SK, Canada; 4 Natural Resources Canada, Canadian Forest Service, Laurentian Forestry Centre, Québec, QC, Canada; 5 Université du Québec en Abitibi-Témiscamingue, Amos, QC Canada; 6 Département des sciences biologiques, Université du Québec à Montréal, Montréal, QC, Canada; 7 Laboratoire de Biotechnologies végétales, É, Faculté des Sciences, Université Ibn Zohr Agadir, Morocco; University of Alberta, CANADA

## Abstract

The mechanistic bases of thermal acclimation of net photosynthetic rate (*A*_*n*_) are still difficult to discern, and the data sets available are scarce, particularly for hybrid poplar. In the present study, we examined the contribution of a number of biochemical and biophysical traits on thermal acclimation of *A*_*n*_ for two hybrid poplar clones. We grew cuttings of *Populus maximowiczii × Populus nigra* (M×N) and *Populus maximowiczii × Populus balsamifera* (M×B) clones under two day/night temperature of 23°C/18°C and 33°C /27°C and under low and high soil nitrogen level. After ten weeks, we measured leaf RuBisCO (*RAR*) and RuBisCO activase (*RARCA*) amounts and the temperature response of *A*_*n*_, dark respiration (*R*_*d*_), stomatal conductance, (*g*_s_), apparent maximum carboxylation rate of CO_2_ (*V*_cmax_) and apparent photosynthetic electron transport rate (*J*). Results showed that a 10°C increase in growth temperature resulted in a shift in thermal optimum (*T*_*opt*_) of *A*_*n*_ of 6.2±1.6°C and 8.0±1.2°C for clone M×B and M×N respectively, and an increased *A*_*n*_ and *g*_s_ at the growth temperature for clone M×B but not M×N. RuBisCO amount was increased by N level but was insensitive to growth temperature while *RARCA* amount and the ratio of its short to long isoform was stimulated by the warm condition for clone M×N and at low N for clone M×B. The activation energy of apparent *V*_*cmax*_ and apparent *J* decreased under the warm condition for clone M×B and remained unchanged for clone M×N. Our study demonstrated the involvement of both *RARCA*, the activation energy of apparent *V*_*cmax*_ and stomatal conductance in thermal acclimation of *A*_*n*_.

## Introduction

**Fig 1:**
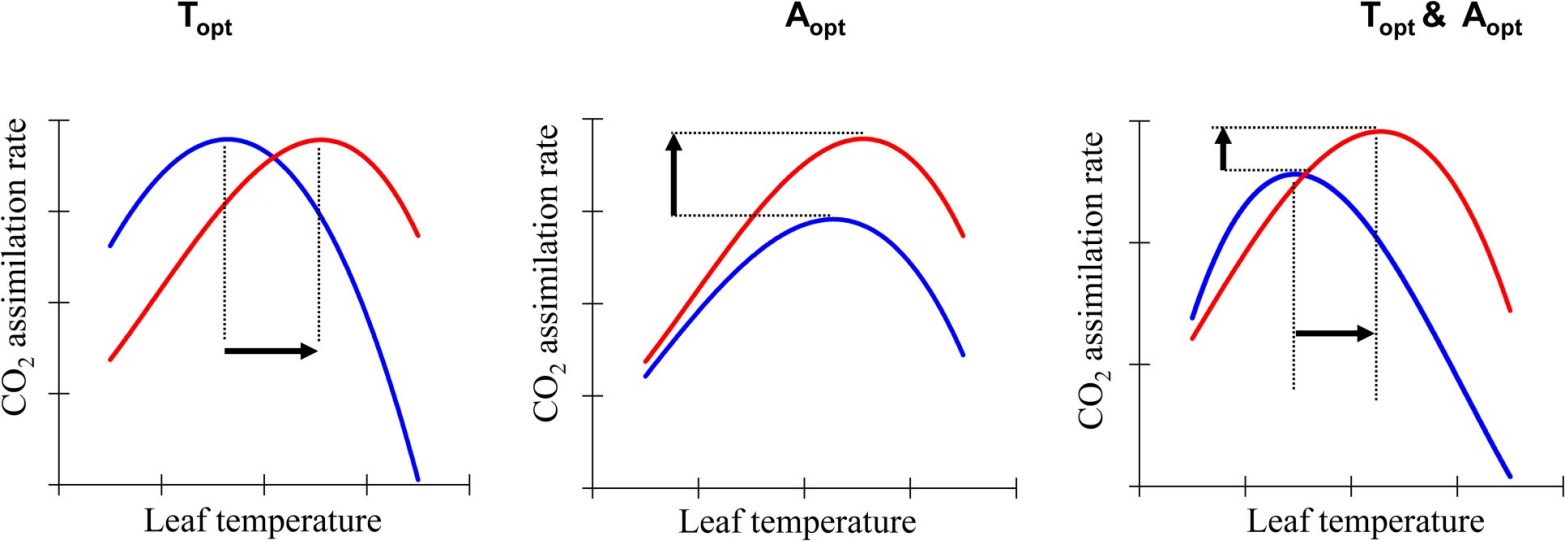
Illustration of scenarios of thermal acclimation of photosynthetic rate. Adapted from Way and Yamori [6]). Blue and red curves indicate the growth under cool and warmer temperature, respectively.

Global warming may lead to a significant reduction of forest productivity through a decrease in net assimilation rate of CO_2_ [1, 2]. Plant physiological processes including photosynthetic rate (*A*_*n*_) and dark respiration (*R*_d_) are strongly temperature-dependent, and their acclimation may help trees maintain a normal growth when temperature shifts from optimum to warm [2–4]. Thermal acclimation of *A*_*n*_ is achieved through adjustments of morphological, biochemical and biophysical components of photosynthesis which may occur via (i) a shift of the thermal optimum of *A*_n_ (*T*_*opt*_) toward the new growth temperature (Fig 1) (ii) an increase or a maintenance of the photosynthetic rate at *T*_*opt*_ (*A*_*n_opt*_) at warmer growth temperatures (iii) a shift in both *A*_*n_opt*_ and *T*_*opt*_. These shifts would result in an increase or maintenance of the photosynthetic rate respective to growth temperature (*A*_*n*_*_*_*growth*_) [5–7]. The mechanisms involved in thermal acclimation of photosynthesis are still difficult to discern and may differ among populations or species from sites with different temperature regimes [8].

Photosynthetic processes that might be subject to acclimation include (i) the reference values (at 25°C) of maximum carboxylation rate (*V*_cmax_^25^) and maximum electron transport rate (*J*_max_^25^), ii) the temperature response of both *V*_cmax_ and *J*_max_ (activation and deactivation energy) and (iii) the temperature response of stomatal and mesophyll conductance [5–7, 9].

Leaf nitrogen (N) might be a limiting factor of carbon assimilation processes and hence plant growth and survival [10, 11], as most of the leaf nitrogen is allocated to proteins involved in light harvesting, Calvin-Benson cycle and electron transfer along thylakoid membranes [12, 13]. Leaf nitrogen content is generally deficient in temperate and boreal regions and has been shown to decrease in response to increasing growth temperature [14–16]. A decrease in leaf N in response to increasing growth temperature may result in a decrease of RuBisCO content [16]. This has been proposed as an explanation of the commonly observed decrease in *V*_cmax_ at temperatures above the optimum and the resulting lack of thermal acclimation of *A*_*n*_ [16, 17]. On the other hand, Yamori et al.’s [18] found that photosynthesis temperature response of several C_3_ plants was generally RuBP carboxylation-limited above the *T*_*opt*_ at low leaf nitrogen content while, under high N level, it shifted to a limitation by RuBP regeneration. However, the effect of temperature on the limiting steps of *A*_*n*_ (*V*_cmax_ *vs*. *J*_max_) may depend on the response of CO_2_ conductance (*g*_*s*_ and *g*_*m*_) as well [19–22]. Moreover, RuBisCO-related effect on *A*_*n*_ at above-optimal temperature may depend on the plasticity of *J*_max_^25^ to *V*_*cmax*_^*25*^ ratio. From this perspective, this may be applicable only for cold-adapted plant species, which are characterized by a higher *J*_max_^25^ to *V*_cmax_^25^ ratio and low or lack of its adjustment in response to both N level and growth temperature [19, 23]. Weston et al.’s [24] did not observe any change in RuBisCO concentration for two genotypes of *Acer rubrum* grown under hot and optimal temperatures. Then, more research is needed to unravel the multiple factors involved in the response of carbon assimilation to above-optimal temperatures. In fact, it has been proven that *V*_cmax_ does not only depend on RuBisCO concentration but also on its activation state (inhibited/activated) [2, 25, 26]. The activation state of RuBisCO is regulated by the RuBisCO activase, a heat-labile enzyme using energy via ATP hydrolysis to release inhibitors from the active site of RuBisCO [26–28]. A decrease in RuBisCO activase activity has been documented as a primary cause of reducing RuBisCO activity and then photosynthetic performance in response to increasing growth temperature [26, 28, 29]. RuBisCO activase is a stromal protein existing in two isoforms of 41–43 kDa (short isoform) and 45–46 kDa (long isoform) that arise from one single gene with an alternatively spliced transcript or from two separate genes. Still, the specific physiological role of a given isoform with respect to heat stress is generally not understood. Recent studies from herbaceous species demonstrated an increase in the two RuBisCO activase forms or a shift in the balance between them when plants were exposed to temperature above 30°C [7, 24, 30–32].

Here we used *Populus*, a model tree in forestry to study the physiological thermal acclimation because of its commercial and environmental importance in the northern hemisphere and its fast growth rate. Information on the response of photosynthesis to higher temperature for tree species is limited in general, and previous studies conducted on *Populus balsamifera* [33], *Populus tremuloides* [34], *Populus nigra* [35], *Populus grandidentata* [15] and *Populus deltoides × nigra* [36] found little evidence of a thermal acclimation of *A*_*n*_ to increasing temperatures. Nevertheless, little research focused on the physiological and molecular mechanisms underlying the observed thermal acclimation of trees. The objective of the present study was to examine to what extent leaf nitrogen, RuBisCO and RuBisCO activase content are involved in thermal acclimation of photosynthetic activity in hybrid poplars. We tested two hypotheses: (1) Leaf N and RuBisCO amounts *per se* are not involved in thermal acclimation of *A*_*n*_. (2) The increase of the RuBisCO activase and or differential expression of its isoforms under warm conditions contribute to thermal acclimation of *A*_*n*_.

## Methodology

### Plant material and growth conditions

This experiment was conducted in greenhouses and growth chambers at Université Laval, Québec, Canada, from January to May 2017. Dormant cuttings of two hybrid poplar clones: M×N (*Populus maximowiczii × Populus nigra*) and M×B (*Populus maximowiczii × Populus balsamifera*) were provided by the Québec’s Ministère des Forêts, de la Faune et des Parcs (MFFP) from the forest nursery of Berthier (Berthierville, Québec, Canada) during early January after chilling needs were met. These clones were recommended by MFFP for the south of Quebec. Cuttings were planted in 2 L pots filled with peat/vermiculite substrate (v/v = 3/1) and placed in two greenhouses where day/night temperatures were 23°C/18°C and 33°C/27°C. Plants were grown under a Photosynthetically-active Photon Flux Density (*PPFD*) ranging between 400 and 700 μmol m^−2^ s^−1^, a relative humidity of 65% and a 8/16 h dark/light photoperiod using 400 W metal halide lamps. Cuttings were irrigated daily to maintain full soil field capacity. After four weeks, for better control of growth conditions (mainly temperature and relative humidity), pots were transferred to growth chambers (model PGW 36, Conviron, Winnipeg, Canada) under a split-split-plot layout; the Temperature × Clone as the first split and Nitrogen level as the second split. The same environmental parameters as in greenhouses were used, except for PPFD which was kept at a constant rate of 500 μmol m^−2^ s^−1^ during day time. In each growth chamber, half of the plants (n = 18) were randomly assigned to receive a low-nitrogen fertilization treatment (5 mM, LN) while the other half received a high-nitrogen (20 mM, HN). Nitrogen was added, every week, using (20N-20P-20K) fertilizer dissolved in distilled water. Plants (n = 72; 2 growth temperatures × 2 nitrogen levels × 2 hybrid poplar clones × 9 replicates) were allowed to acclimate to respective growth conditions for six weeks before measurements were taken. Pots were moved within each chamber every third day to eliminate any position-related bias.

### Gas exchange measurements

After ten weeks of growth, leaf-level gas exchange was measured on the 4^th^ fully expanded leaf from the top of each plant using two cross-calibrated portable open-path gas-exchange systems (Li-6400, Li-Cor Inc., Lincoln NE), equipped with a 2×3 cm broadleaf chamber (Li-6400-40, Li-Cor Inc). The measurements were made on 24 plants in total (3 replicates × 2 clones × 2 temperatures × 2 N levels). Given the limited control capacity of LI-6400 system on leaf temperature in the cuvette (*T*_*leaf*_ can be set to ± 6°C of the ambient temperature), measurements were performed in a growth chamber under controlled temperature and relative humidity. Growth chamber temperature was set manually to desired *T*_*leaf*_ allowing an effective and quick easy adjustment over the 10–40°C range and an exposure of the whole plant to the targeted temperature.

The temperature was increased from 10°C to 40°C with 5°C increment and plants were allowed to acclimate for at least 20 min to each step. At each temperature, we measured dark respiration (*R*_*d*_) followed by *A-C*_*i*_ response curve records with a 10-minutes period between *R*_*d*_ and *A-C*_*i*_ respected to allow complete opening of stomata. *A-Ci* response curves were recorded at each temperature after at least 10 min of steady state at ambient CO_2_ partial pressure *C*_*a*_ = 400 μmol mol^-1^ and a saturating *PPFD* = 800 μmol m^-2^ s^-1^. The saturated *PPFD* was determined from measured *A-Q* curve on 3 plants from each Clone × Growth T° combination at 25°C. Thereafter, the reference CO_2_ (*C*_*a*_) was changed in the following order: 400, 350, 300, 200, 100, 50, 400, 500, 600, 800, 900, 1000, 1200, 1400, and 1600 μmol mol^-1^. Values were recorded based on the stability of photosynthesis, stomatal conductance (*g*_*s*_), CO_2_ and water vapour concentration. The vapour pressure difference (*VPD*) during measurement varied from 0.5 to 3.2 KPa from low to high temperature. At high temperature, the VPD was lowered as much as possible by maintaining the relative humidity (RH) at 70% inside the growth chamber. At low temperature, RH was maintained at 50% to maintain VPD as high as 0.5 KPa. For each sample, data required were collected generally within one or two days (10–14 h). The list of abbreviations and symbols are given in Table 1.

**Table 1:** List of abbreviations.

Symbol	Definition	Unit
*A*_*c*_	RuBP-saturated CO_2_ assimilation rate	*μ*mol CO_2_ m^-2^ s^-1^
*A*_*n*_*_*_*growth*_	Photosynthetic rate at growth temperature	*μ*mol CO_2_ m^-2^ s^-1^
*A*_*n*_	Net CO_2_ assimilation rate	*μ*mol CO_2_ m^-2^ s^-1^
*A*_*j*_	RuBP-limited CO_2_ assimilation rate	*μ*mol CO_2_ m^-2^ s^-1^
*A*_*n_opt*_	Photosynthetic rate at *T*_*opt*_	*μ*mol CO_2_ m^-2^ s^-1^
*C*_*a*_	Atmospheric CO_2_ concentration	μmol mol^-1^
*C*_*i*_	intercellular CO_2_ concentration	μmol mol^-1^
*E*_*a*_	Activation energy	KJ mol^-1^
*E*_*d*_	Energy of deactivation	KJ mol^-1^
*g*_*s*_	Stomatal conductance	mol H_2_O m^-2^ s^-1^
*J*	Electron transport rate	*μ*mol e^-^ m^-2^ s^-1^
*J*_*max*_^*25*^	Maximal electron transport rate at leaf temperature of 25°C	*μ*mol e^-^ m^-2^ s^-1^
*J*_*max*_^*25*^:*V*_*cmax*_^*25*^	Ratio of maximal electron transport to maximal carboxylation rate at leaf temperature of 25°C	
*N*_*area*_	Leaf nitrogen in area basis	g m^-2^
*O*	Partial atmospheric pressure of O_2_	mmol mol^-1^
*PPFD*	Photosynthetically-active Photon Flux Density	*μ*mol m^-2^ s^-1^
*SLA*	Specific leaf area	cm^2^ g^-1^
*R*_*day*_	Non-photorespiratory mitochondrial respiration in the light	*μ*mol CO_2_ m^-2^ s^-1^
*R*_*d*_	Dark respiration	*μ*mol CO_2_ m^-2^ s^-1^
*R*_*d*_^*10*^	*R*_*d*_ at leaf temperature of 10°C	*μ*mol CO_2_ m^-2^ s^-1^
*T*_*opt*_	Thermal optimum	°C
*K*_*c*_	Michaelis–Menten constants of RuBisCO for CO_2_	μmol mol^-1^
*K*_*o*_	Michaelis–Menten constants of RuBisCO for O_2_	mmol mol^-1^
*Q*_*10*_	Rate of change in *R*_*d*_ with a 10°C increase in temperature	
Γ^*^	CO_2_ compensation point in the absence of mitochondrial respiration	μmol mol^-1^
*Α*	Efficiency of light energy conversion	
*V*_*cmax*_	Maximal carboxylation rate	*μ*mol CO_2_ m^-2^ s^-1^
*V*_*cmax*_^*25*^	Maximal carboxylation rate at leaf temperature of 25°C	*μ*mol CO_2_ m^-2^ s^-1^

### Estimation of gas exchange variables

The photosynthetic capacity variables, *V*_cmax_ and *J*_max_, were estimated from gas-exchange by fitting the *A-C*_*i*_ curve with the biochemical model of C_3_ [37], assuming infinite mesophyll conductance (*g*_m_). In fact, the estimation of *g*_m_ from *A-*Ci is very challenging as it depends on the number of data points on the *A-*Ci curve and goodness-of-fit of the curve which is difficult to achieve at high and low temperatures. In this experiment, we tried to estimate *g*_m_ from *A*-Ci curves following Ethier et al.’s [38] and Miao et al.’s [39] without success as about 45% of them gave non-meaningful estimates.

The model was thus fitted using non-linear regression techniques (Proc NLIN, SAS) following Dubois et al.’s [40]. Briefly, the net assimilation rate (*A*_*n*_) is given as:

$$A_{n}=min\{A_{c},A_{j}\}$$ (1)

$$A_{c}=V_{cmax}\frac{\left(C_{i}-\Gamma^{*}\right)}{C_{i}+K_{c}(1+O/K_{o})}-R_{day}$$ (2)

$$A_{j}=J\frac{C_{i}-\Gamma^{*}}{4(C_{i}+{2\Gamma }^{*})}-R_{day}$$ (3)

$$J=\frac{\alpha Q}{\sqrt{1+{\left(\frac{\alpha Q}{J_{max}}\right)}^{2}}}$$ (4)

where *V*_cmax_ is the apparent maximum rate of carboxylation (*μ*mol CO_2_ m^-2^ s^-1^), *O* is the partial atmospheric pressure of O_2_ (mmol mol^-1^)_,_ Γ^*^ is the CO_2_ photo-compensation point in the absence of mitochondrial respiration, *R*_*day*_, is mitochondrial respiration in the light (*μ*mol CO_2_ m^-2^ s^-1^), *C*_*i*_ is the intercellular (substomatal) concentration of CO_*2*_ (μmol mol^-1^), *K*_*c*_ (μmol mol^-1^) and *K*_*o*_ (mmol mol^-1^) are the Michaelis–Menten constants of RuBisCO for CO_2_ and O_2,_ respectively, *J* is the apparent rate of electron transport (*μ*mol e^-^ m^-2^ s^-1^), *J*_max_ is the apparent maximum rate of electron transport (*μ*mol e^-^ m^-2^ s^-1^), *Q* is the incident *PPFD* (*μ*mol m^-2^ s^-1^), *α* is the efficiency of light energy conversion (0.18) which represents the initial slope of the photosynthetic light response curve [39]. The values at 25°C used for *K*_*c*_, *K*_*o*_ and Γ^*^ were 272 μmol mol^-1^, 166 mmol mol^-1^ and 37.4 μmol mol^-1^, respectively and their temperature dependency was from Bernacchi *et al*.’s [41]. Most of the *A-C*_*i*_ curves at 35°C and 40°C measured for low nitrogen level at 23°C failed to converge and estimates of apparent *V*_*cmax*_ and apparent *J* could not be obtained.

### Characterization of the temperature responses of gas exchange variables

Photosynthesis temperature response curves were fitted individually with a quadratic model following Battaglia et al.’s [42]:

$$A_{n}(T)=A_{opt}-b{\left(T-T_{opt}\right)}^{2}$$ (5)

where *A*_*n*_*(T)* is the photosynthetic rate at temperature T in°C, *A*_*n_opt*_ is the photosynthetic rate at the temperature optimum (*T*_*opt*_), and the parameter b describes the spread of the parabola.

*A*_*n*_*_*_*growth*_ was then estimated using the obtained parameters from Eq (5) for each curve. The daytime temperature was used as growth temperature given the uncertainty regarding the effect of nighttime temperature on *A*_*n*_.

Dark respiration temperature response curves were fitted with a model in Eq (6) to estimate the *Q*_*10*_ (the change in respiration with a 10°C increase in temperature) following Atkin et al.’s [3]:

$$R_{d}(T)={R_{d}}^{10}{Q_{10}}^{\left[(T-10)/10\right]}$$ (6)

where *R*_*d*_^*10*^ is the measured basal rate of *R*_*d*_ at the reference temperature of 10°C.

The responses of apparent *V*_*c*max_ and apparent *J* to leaf temperature were fitted using the following two models (Eqs (7) and (8)) depending on the presence or not of deactivation above thermal optimum following Medlyn et al.’s [4]:

$$f(T_{k})=e^{\left(c-\frac{E_{a}}{RT}\right)}$$ (7)

$$f(T_{k})=k_{opt}\frac{E_{d}\;{exp}^{\left[\frac{E_{a}(T_{k-}T_{opt})}{T_{k}RT_{opt}}\right]}}{E_{d}-E_{a}[1-{exp}^{\left(\frac{E_{d}(T_{k}-T_{opt})}{T_{k}RT_{opt}}\right)}]}$$ (8)

where *E*_*a*_ is the activation energy, *E*_*d*_ is the energy of deactivation, *K*_*opt*_ is the apparent *V*_*cmax*_ or apparent *J* at the temperature optimum (*T*_*opt*_). *E*_*d*_ was fixed at 200 KJ mol^-1^ [4] to reduce the number of estimated parameters to three.

### Specific leaf area and leaf nitrogen

Leaves used for gas exchange measurements were collected and immediately placed in dry ice before being stored at -20°C and processed within a week for protein extraction. The extracts were kept at -80°C and dosage of proteins (RuBisCO and RuBisCO activase) was done once all samples were extracted. Symmetric leaves (by the stem) were also collected to measure projected area with WinSeedle (Version 2007 Pro, Regent Instruments, Québec, Canada). Samples were then oven-dried for 72h at 56°C, and their dry mass determined. Specific leaf area (*SLA*) was calculated as the ratio of the projected leaf area (cm^2^) to the leaf dry mass (g). Later, leaves were ground separately and N content determined at Université Laval using a LECO elemental analyzer (LECO Corporation, St Joseph, MI, USA).

### Extraction and dosage of RuBisCO and RuBisCO activase

Proteins were extracted from frozen leaves at -20°C within less than one week after leaf harvesting following the method outlined in Yamori and von Caemmerer [28]. Briefly, 100 mg of leaves were initially ground in liquid nitrogen using a mortar and pestle. Proteins were extracted on ice using a protein extraction buffer containing 50 mM Hepes-KOH pH 7.8, 10 mM MgCl_2_, 1 mM EDTA, 5 mM DTT, 0.1% Triton X100 (v/v) and protease inhibitor cocktail (Roche). The extracts were kept at -80°C, and once all samples were extracted, the solutions were centrifuged at 16,000g for 1 min followed by determination of the concentration of total soluble proteins (TSP) in the supernatant by the Bradford method [43].

After dosage, 4× sample buffer (250 mM Tris–HCl, pH 6.8, 40% glycerol, 8% SDS, 0.2% Bromophenol-blue, 200 mM DTT) was added to proteins extracts, heated at 100°C for 5 min and then centrifuged at 16,000 g for 5 min. After cooling to room temperature, a volume representing 20 μg of total TSP extract of each sample was loaded onto 12% SDS-polyacrylamide gel electrophoresis (SDS-PAGE). The electrophoresis was carried out at room temperature at a constant voltage (120 V). Following SDS-Page, the proteins were transferred to a nitrocellulose membrane (Life Sciences, Mississauga, Canada) for western blot.

Blots were incubated with 5% non-fat milk in TBST (50 mM Tris, pH 7.5, 150 mM NaCl, 0.1% Tween-20) for 60 min, the membranes were washed twice with TBST and incubated with antibodies against RuBisCO (Agrisera AB, Vännäs, Sweden) or RuBisCO activase (Agrisera AB, Vännäs, Sweden) at room temperature for 60 min. Membranes were washed three times with TBST for 10 min and incubated with secondary antibodies peroxidase-conjugated (Goat Anti-Chicken (Abcam) for RuBisCO and Goat Anti-Rabbit (Abcam) for RuBisCO activase) during 60 min at room temperature. Blots were washed with TBST three times and developed with the ECL system using Odyssey Infrared Imaging System (Li-COR, Biosciences). Images were analyzed using ImageJ [44] to determine the band densities of each sample. The RuBisCO, RuBisCO activase and its two isoforms concentration were expressed as relative to the sample representing the highest density (RAR and RARCA respectively) [31, 45, 46].

### Statistical analysis

Three-way analysis of variance was performed to test the effect of growth temperature, clone and nitrogen level on response variables using MIXED procedure of SAS (SAS Institute, software version 9.4, Cary, NC, USA). We used Proc Glimmix for response variables (apparent *V*_cmax_^25^, apparent *J*_*max*_^25^ and *E*_*a*_) which did not meet the assumptions of residual normality and homoscedasticity even with transformations. Means were compared by the adjusted Tukey method and differences were considered significant if *P* ≤ 0.05.

## Results

### Temperature response of *A*_*n*_ and *R*_*d*_

**Fig 2:**
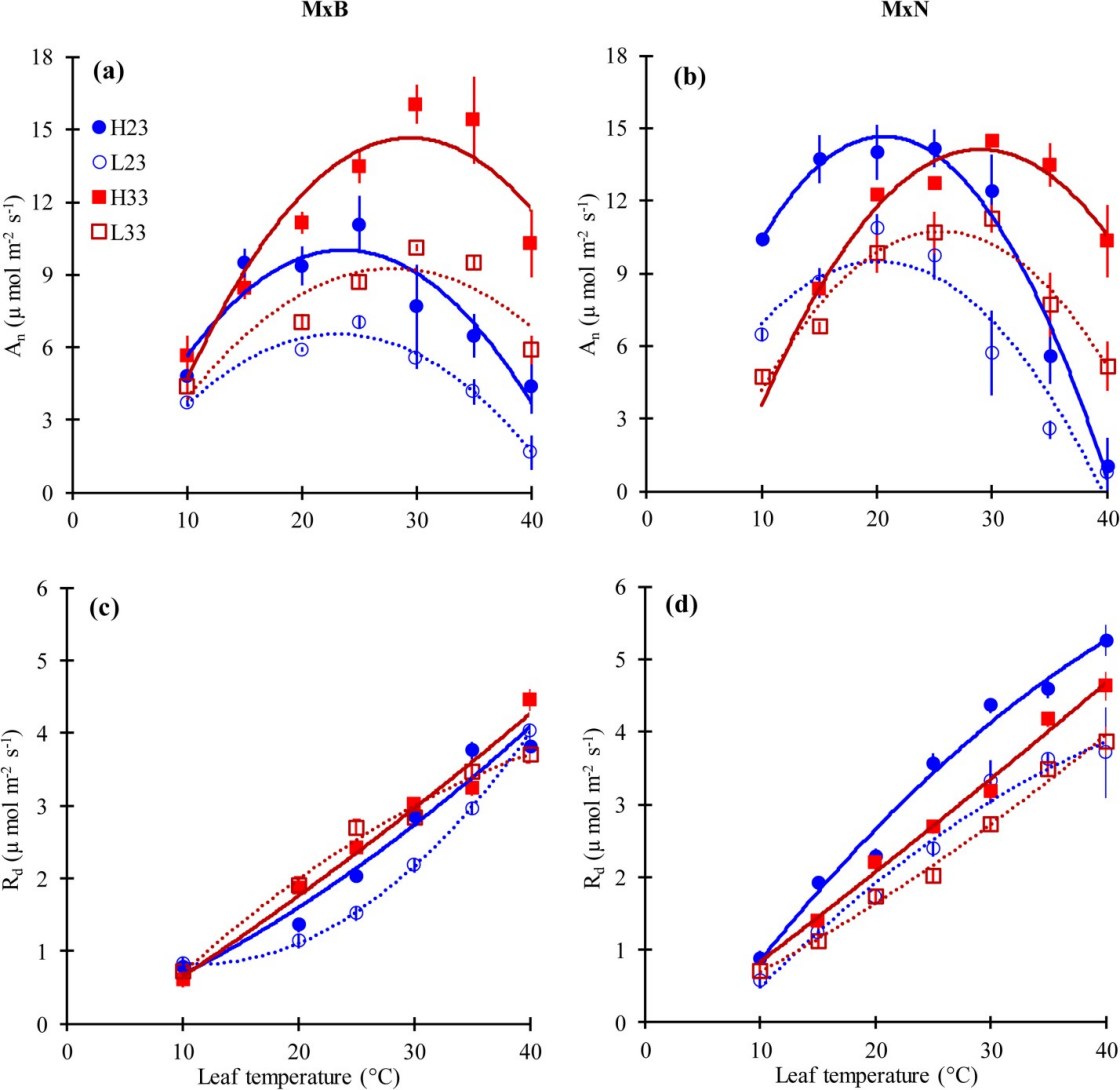
The response of net photosynthesis (*A*_*n*_) and dark respiration (*R*_*d*_) to leaf temperature for hybrid poplar clone M×B (a, c) and clone M×N (b, d) grown under two temperatures and two nitrogen levels. H23 and L23 are treatments of high and low nitrogen level respectively at an ambient day temperature of 23°C; H33 and L33 are treatments of high and low nitrogen level at 33°C ambient day temperature. Data are represented by means ± SE (n = 3). *P* value and *R*^*2*^ of curves are given in S2 Table.

The temperature response curves of net photosynthesis under saturating irradiance (*A*_*n*_) were nicely fitted with a parabolic function (Fig 2A and 2B). Thermal optima (*T*_*opt*_) of *A*_*n*_ differed between clones and increased in response to growth temperature. Low nitrogen level constrained the adjustment of *T*_*opt*_ for clone M×N but not M×B (Table 2). Also, *T*_*opt*_ was below growth temperature except for clone M×B at 23°C. The two hybrid poplar clones showed different trends regarding *A*_*n*_ at *T*_*opt*_ (*A*_*n_opt*_) which increased with increasing growth temperature for clone M×B and remained unaffected for clone M×N under high N treatment. *A*_*n*_*_*_*growth*_ had a similar trend as *A*_*n_opt*_ in response to growth temperature and N level. Both *A*_*n*_*_*_*growth*_ and *A*_*n_opt*_ declined at low N level for both clones (Table 2).

**Table 2:** Means (±SE) of thermal acclimation-related traits of two hybrid poplar clones (M*×*B and M*×*N) grown at day/night temperature of 23/18°C and 33/27°C under high (HN) and low (LN) nitrogen levels (n = 3).

	Clone M×B				Clone M×N			
	23°C		33°C		23°C		33°C	
	HN	LN	HN	LN	HN	LN	HN	LN
***T***_***opt***_***(A***_***n***_***)***	23.1 (1.2)bc	24.1 (1.2)b	30.3 (1.3)a	29.3 (1.3)a	20.5 (1.2)c	19.7 (1.3)c	30.1 (1.3)a	26.1 (1.3)b
***A***_***n_opt***_	10.1 (0.8)b	7.1(0.8)d	14.9 (0.8)a	9.3 (0.8)cb	15.1 (0.8)a	9.1 (0.8)c	14.2 (0.8)a	10.9 (0.8)b
***A***_***n***_***_***_***growth***_	10.6(1.0)b	6.9(0.9)c	13.9(1.1)a	8.9(1.1)c	14.9(1.1)a	9.6(0.9)c	13.4(1.1)a	9.5(1.1)c
***R***_***d***_^***25***^	2.02(0.2)c	1.51(0.2)d	2.41(0.2)bc	2.61(0.2)b	3.56(0.2)a	2.39(0.2)bc	2.66(0.2)b	2.01(0.2)c
***Q***_***10***_ ***(R***_***d***_***)***	1.9(0.1)b	1.8(0.1)b	2.0(0.1)a	1.9(0.1)b	2.0(0.1)a	2.2(0.1)a	1.8(0.1)b	1.8(0.1)b
***g***_***s_growth***_	0.16(0.01)c	0.17(0.01)bc	0.26(0.01)a	0.20(0.01)b	0.20(0.01)b	0.16(0.01)c	0.18(0.01)bc	0.13(0.01)d
***T***_***opt***_***(***apparent ***V***_***cmax***_***)***	33(1.5)	-	NA	NA	34(1.2)	-	NA	NA
***E***_***a***_***(***apparent ***V***_***cmax***_***)***	75 (3)a	-	49(3)b	57(3)b	58(3)b	-	54(3)b	55(3)b
***T***_***opt***_***(***apparent ***J)***	34 (1.9)	-	NA	NA	30(1.1)	-	NA	NA
***E***_***a***_***(***apparent ***J)***	46(2)a	-	32(2)b	28(2)b	34(2)ab	-	28(2)b	33(2)b
apparent ***J***_***max***_^***25***^: apparent ***V***_***cmax***_^***25***^	2.43(0.1)ab	1.68(0.15)c	1.53(0.15)c	1.81(0.19)bc	2.68(0.15)a	2.01 (0.15)bc	2.42(0.19)ab	1.95(0.16)b
***SLA***	172(7)a	132(7)cd	154(8)b	123(7)d	143(7)b	141(7)bc	136(8)c	112(8)e
***N***_***area***_	1.3(0.1)b	0.8(0.1)d	1.3(0.1)b	0.8(0.1)d	1.9(0.1)a	1.1(0.1)c	1.4(0.1)b	1.1(0.1)c

Within rows, means followed by the same letter do not differ significantly at α = 0.05 based on Tukey’s test. ANOVA results are given in S1 Table.

The two hybrid poplar clones had a different thermal response of dark respiration (*R*_*d*_) (Fig 2C and 2D). The rate of *R*_d_ (*R*_*d*_^*25*^) decreased by augmenting growth temperature for clone M×N at high N level and increased by augmenting growth temperature for clone M×B at low N level (Table 2). *Q*_*10*_, decreased when growth temperature was increased, irrespective of N level for clone M×N. In contrast, *Q*_*10*_ of clone M×B increased in response to growth temperature raise when N level was high and unchanged at low N level (Table 2).

### Temperature response of apparent *V*_cmax_ *a*nd *J*

**Fig 3:**
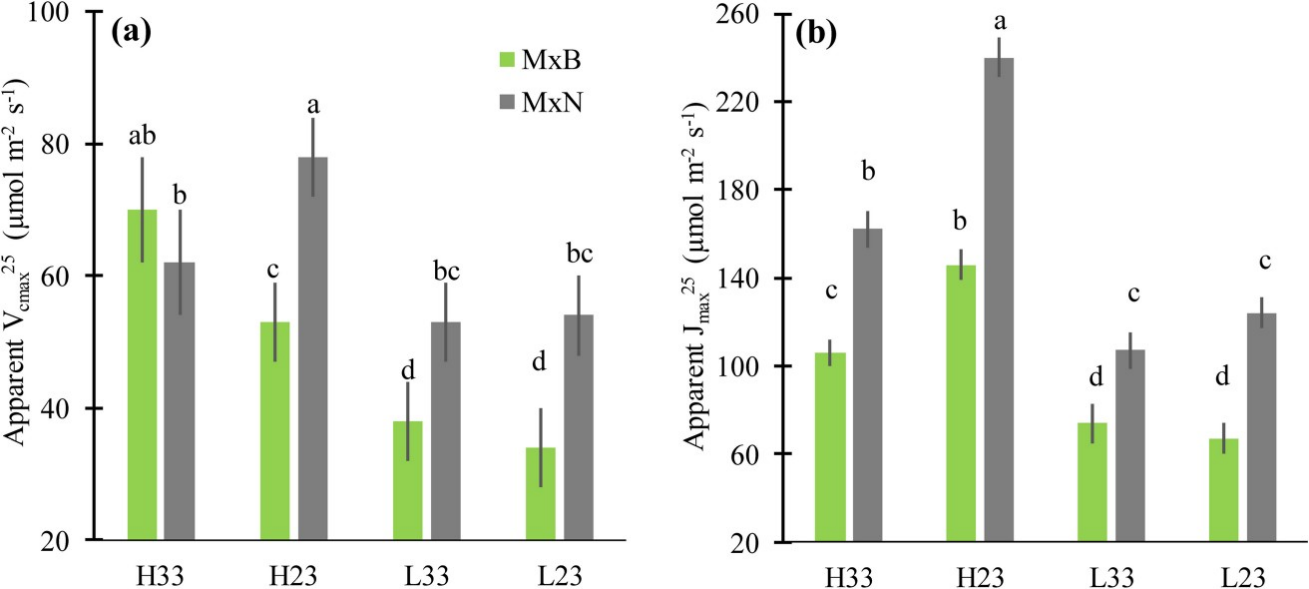
**Apparent maximum carboxylation rate of RuBisCO at leaf temperature of 25°C (*V***_**cmax**_^**25**^**) (a), and apparent maximum electron transport rate at leaf temperature of 25°C (*J***_**max**_^**25**^ **) (b) for two hybrid poplar clones (M×B) and (M×N) grown under two temperatures and two nitrogen levels.** See Fig 2 for abbreviation. Data are represented by means ± SE (n = 3). Means having the same letters are not significantly different at α = 0.05 based on Tukey’s tests.

Apparent *V*_cmax_^25^ was insensitive to growth temperature at low N level for both clones. In contrast, at high N level, apparent *V*_cmax_^25^ increased for clone M×B and decreased for clone M×N when growth temperature was increased (Fig 3). Apparent *J*_max_^25^ decreased with increasing growth temperature for plants growing at high N level and was insensitive to growth temperature at low N level (Fig 3). The ratio apparent *J*_max_^25^: apparent *V*_cmax_^25^ decreased with increasing growth temperature at high N level for clone M×B but not for clone M×N (Table 2). At low N level, apparent *J*_max_^25^: apparent *V*_cmax_^25^ ratio was insensitive to growth temperature.

**Fig 4:**
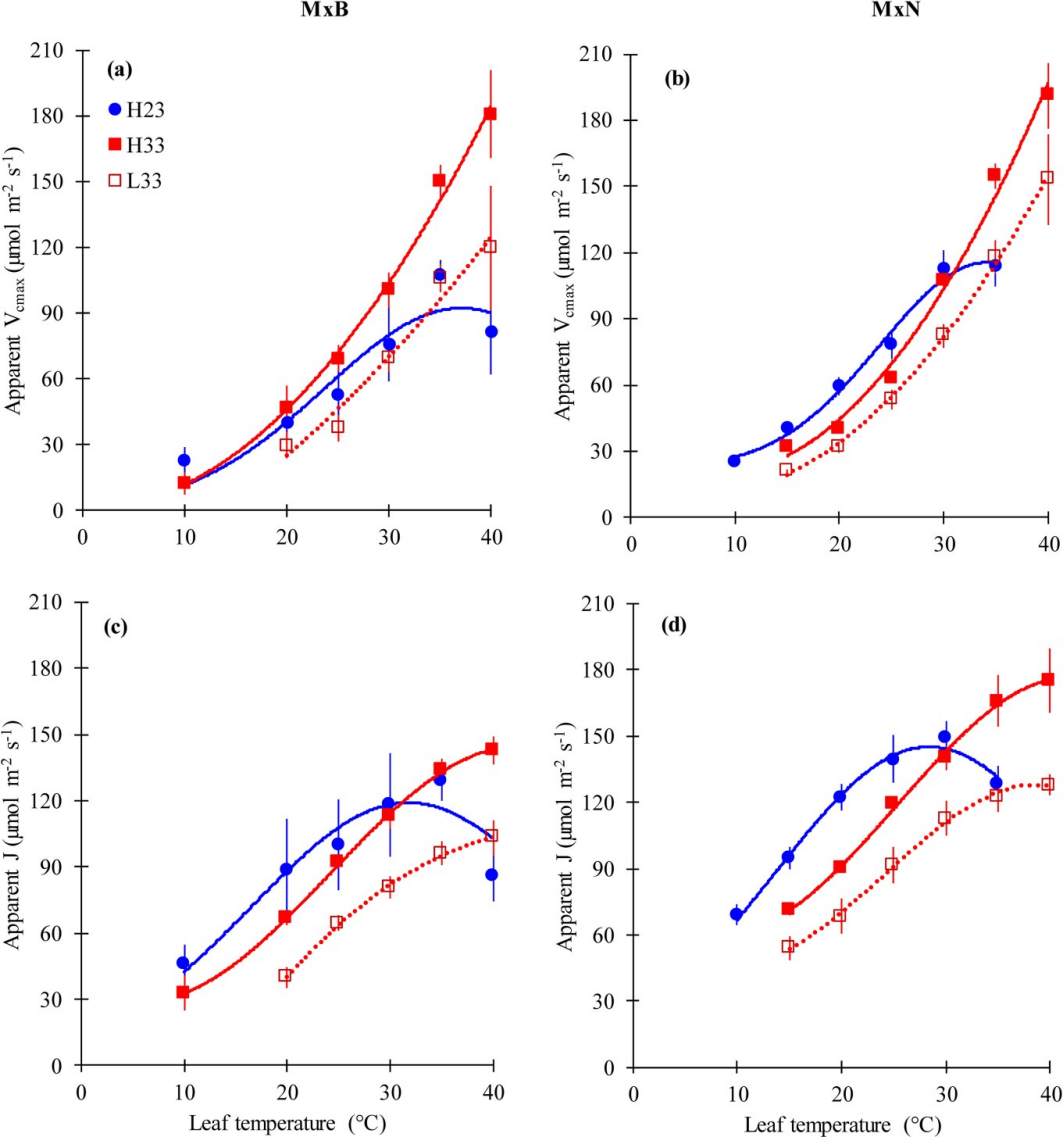
**The temperature dependence of the apparent maximum carboxylation capacity of RuBisCO (*V***_**cmax**_**) and the apparent electron transport rate (*J*) for clone M×B (a, c) and clone M×N (b, d) grown under two temperatures and two nitrogen levels.** See Fig 2 for symbols. L23 treatment was not given for both clones because A-Ci curves at 35 and 40°C failed to converge and estimates of *V*_*cmax*_ and *J* could not be obtained. Data are represented by means ± SE (n = 3). *P* value and *R*^*2*^ of curves are given in S2 Table.

The temperature response curves of apparent *V*_cmax_ and apparent *J* were affected by growth temperature but not by nitrogen level. In fact, at the cooler growth temperature, apparent *V*_cmax_ peaked at 33°C and 34°C (Fig 4; Table 2) and apparent *J* peaked at 34°C and 30°C (Fig 4; Table 2) for clones M×B and M×N respectively. However, apparent *V*_cmax_ and apparent *J* did not show any deactivation at warm temperature (Fig 4). The activation energy (*E*_*a*_) of apparent *V*_cmax_ and *J*, decreased with increasing growth temperature for clone M×B and remained constant for clone M×N (Table 2).

### Temperature response of stomatal conductance (*g*_s_)

**Fig 5:**
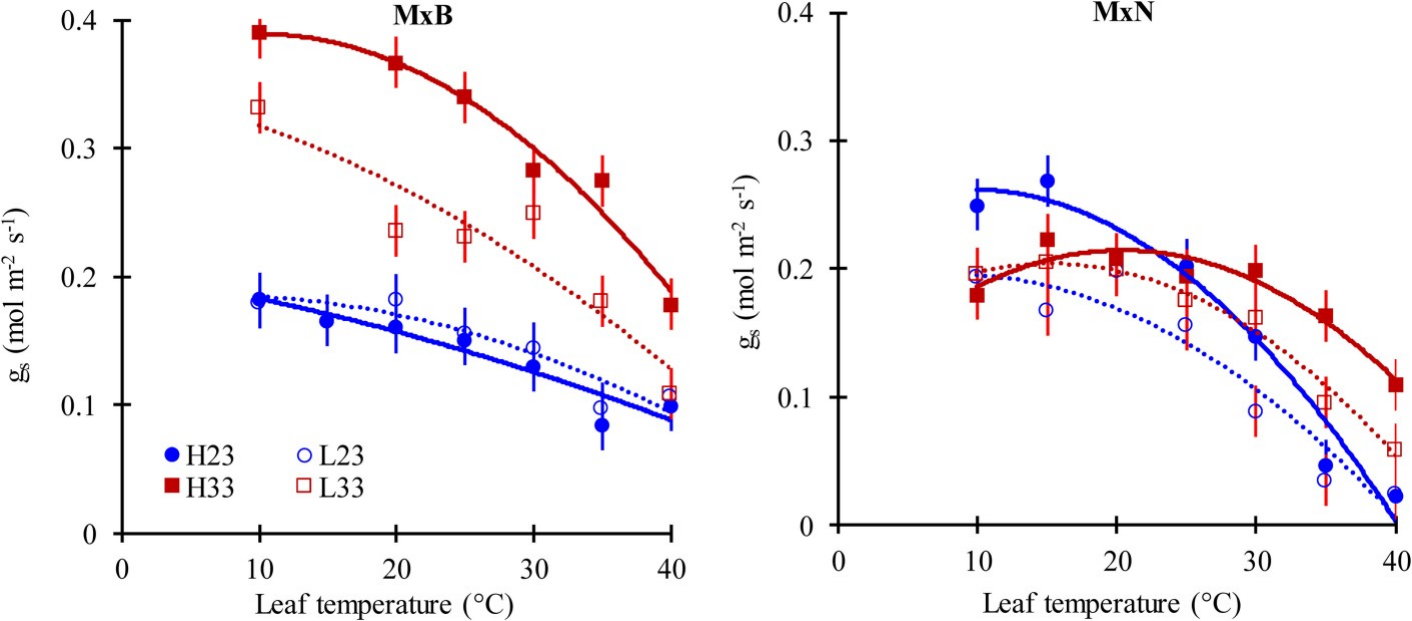
The response of stomatal conductance (*g*_*s*_) to leaf temperature of two hybrid poplar clones (M×B) and (M×N) grown under two temperatures and two nitrogen levels (n = 3). See Fig 2 for symbols. Data are represented by means ± SE (n = 3). *P* value and *R*^*2*^ of curves are given in S2 Table.

*g*_s_ decreased under all treatments and for both clones when *T*_*leaf*_ was increased over the 10–40°C gradient (Fig 5). *g*_s_ at the growth temperature, derived from the *g*_*s*_*-T* response curves (*g*_*s_growth*_) was influenced by both clone and growth temperature. For clone M×B, *g*_*s_growth*_ was 62.5% and 17% higher at warm, compared to cooler growth temperature under high and low nitrogen level respectively (Table 2). Conversely, for clone M×N, *g*_*s_growth*_ was similar among growth temperature at high N level averaging 0.19 mol H_2_O m^−2^ s^−1^ and decreased by increasing growth temperature at low N level (0.16 vs. 0.13 mol H_2_O m^−2^ s^−1^).

### Specific leaf area and leaf nitrogen

Leaf nitrogen content expressed on an area basis (*N*_*area*_) was increased under high N treatment for both clones. Growth temperature impacted negatively *N*_*area*_ of clone M×N only at high N level (Table 2). *SLA* decreased by increasing growth temperature except for clone M×B under low N. Also, *SLA* was greater in HN than LN except for clone M×N at 23°C (Table 2).

### RuBisCO and RuBisCO activase amount

**Fig 6:**
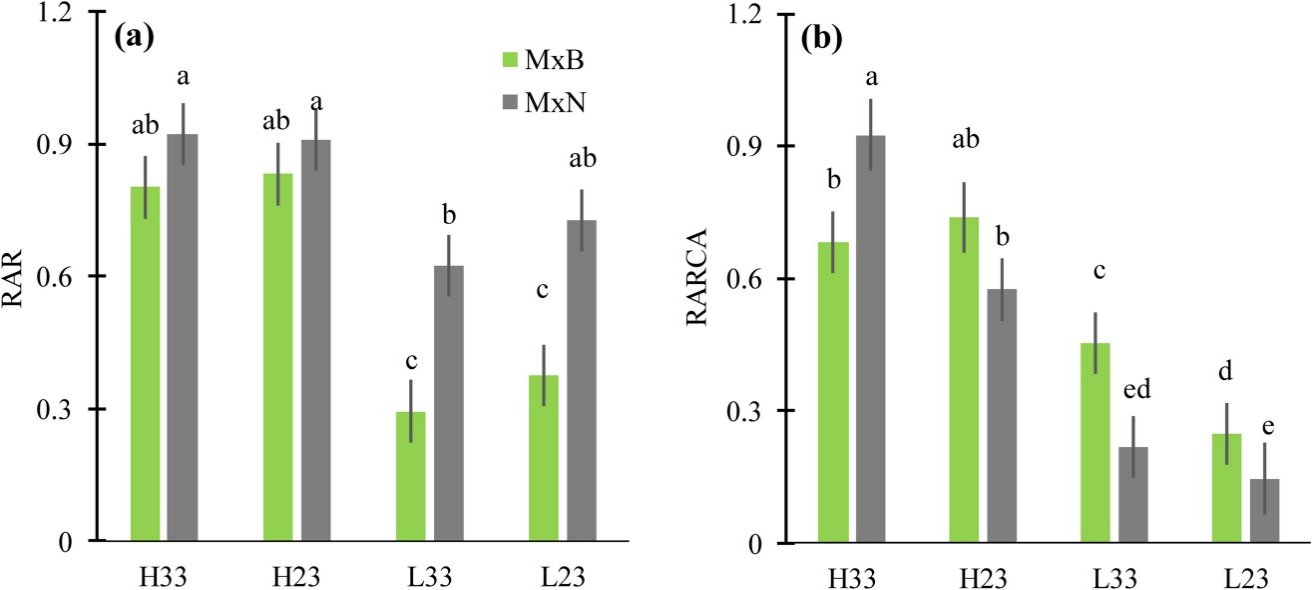
**Relative amounts of RuBisCO (RAR) (a) and RuBisCO-activase (RARCA) (b) measured by western blot for two hybrid poplar clones (M×B) and (M×N) grown under two temperatures and two nitrogen levels (n = 3).** Proteins were extracted from leaves and analyzed by SDS-PAGE. Immunoblots were probed with anti-RuBisCO or anti-RuBisCO activase antibody (S1 Fig). H23 and L23 are treatments of high and low nitrogen level respectively at 23°C ambient daytime temperature; H33 and L33 are treatments of low and high nitrogen level at 33°C ambient daytime temperature. Data are represented by means ± SD (n = 3). Means having the same letters are not significantly different at α = 0.05 based on Tukey’s tests.

**Fig 7:**
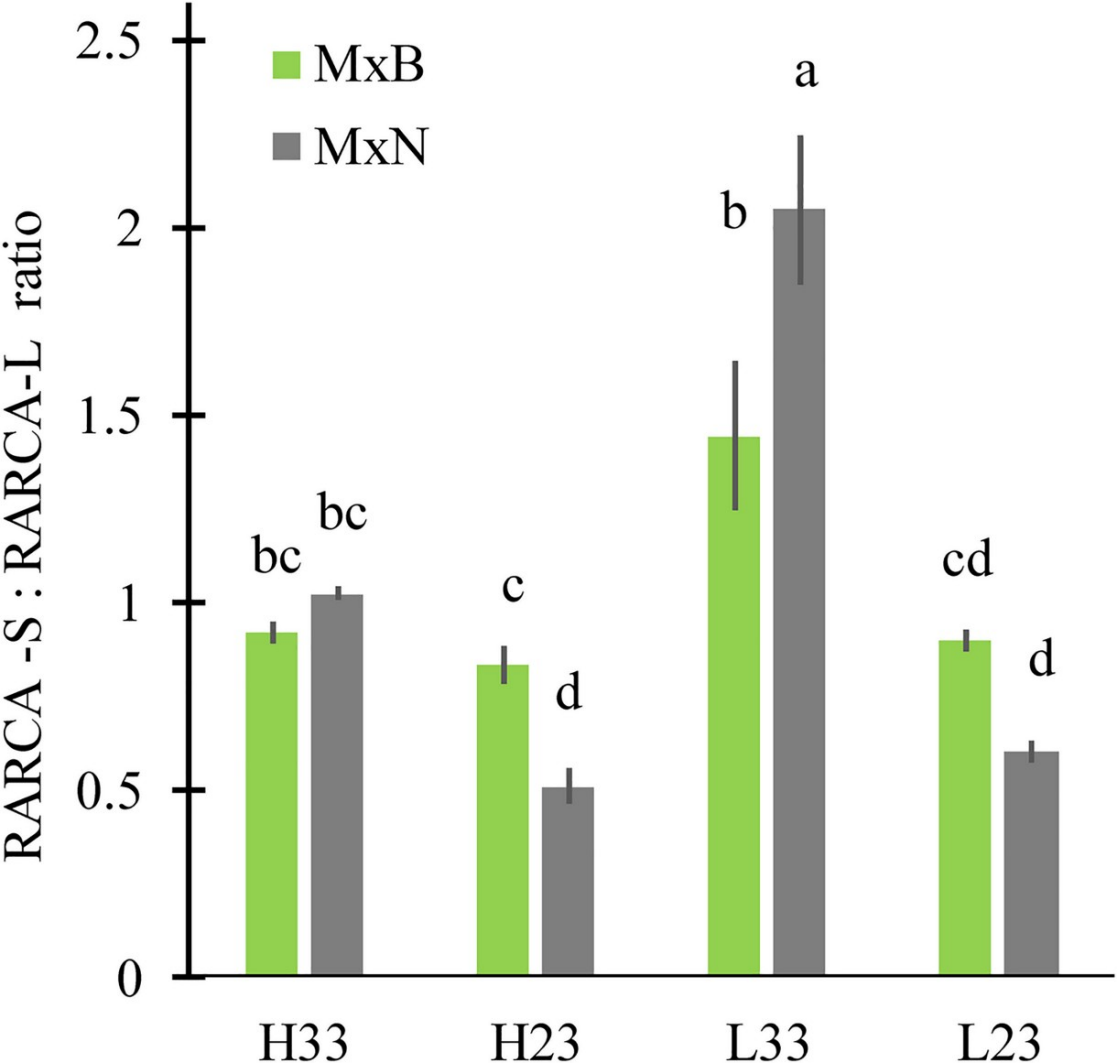
Ratio of short to long isoform of RuBisCO activase of two hybrid poplar grown under two temperatures and two nitrogen levels (n = 3). RCA-S: the short isoform of RuBisCO activase; RCA-L: the long isoform of RuBisCO activase. Data are represented by means ± SE (n = 3). Means having the same letters are not significantly different at α = 0.05 based on Tukey’s test.

The relative amount of RuBisCO (*RAR*) decreased significantly when N level changed from high to low, except for M×N at 23°C (Fig 6A). *RAR* did not change in response to change of growth temperature for both clones (Fig 6A). In addition, at high N level, *RAR* was similar between clones, being around 0.8 on average. At low N level, RAR was two folds higher for clone M×N compared to clone M×B (Fig 6A). Nitrogen enrichment remarkably increased the relative amount of RuBisCO activase (*RARCA*), particularly for clone M×N which had a lower *RARCA* at low N level, compared to M×B (Fig 6B). *RARCA* was stimulated by warmer growth temperature for M×N at high N and for M×B at low N, but no difference was found for the two other clone-N combinations (Fig 6B). More importantly, the ratio of short isoform to large isoform of RuBisCO activase was markedly simulated by warm conditions for clone M×N and only at low N for clone M×B (Fig 7).

## Discussion

### Thermal acclimation of *A*_*n*_ and *R*_*d*_

The two hybrid poplar clones showed a clear thermal acclimation of *A*_*n*_ by adjusting *A*_*n-opt*_ and/or *T*_*opt*_ to growth temperature. This is in accordance with results of [33] on cold and warm ecotypes of *Populus balsamifera* which maintained *A*_*n-opt*_ without an evident change of *T*_*opt*_. We found that *T*_*opt*_ of *A*_*n*_ under warm temperature was identical to mean growth temperature (the average of day time/night-time = 30°C) and was 3°C below the daytime growth temperature (33°C) suggesting a limited acclimation of photosynthesis rate if we assume the latter was unrelated to night-time temperature. So far, studies focusing on night-time temperature effect on *A*_*n*_ are very scarce [47]. *T*_*opt*_ of *A*_*n*_ for clone M×N was lowered by low nitrogen level under warms conditions. The net photosynthetic rates at the growth temperature (*A*_*n*_*_*_*growth*_), a relevant quantitative trait that reflects thermal acclimation of *A*_*n*_ [6, 7], was enhanced in plants grown at the warm temperature for clone M×B and remained unchanged for clone M×N. These results suggest a differential thermal adaptation range of the two hybrid poplar clones which could result from the climate of origin of their parents [33]. In a recent meta-analysis, Kumarathunge et al.’s [48] reported that the modulation of *T*_*opt*_ and *A*_*opt*_ in response to the change in growth temperature was driven by acclimation and, to a lesser extent, by local genetic adaptation to the climate of origin.

Thermal acclimation of *R*_d_ is very common for C_3_ plants and several studies reported a downshift in the rate of *R*_*d*_ (so-called Type II acclimation) and a decrease of *Q*_*10*_ (so-called Type I acclimation) in response to warmer temperatures [49, 50] but few studies on *Populus* exist in this regard [33, 36, 51, 52]. In accordance with the findings of Tjoelker et al.’s [52] for *Populus tremula*, we found substantial Type I and II acclimation of *R*_*d*_ to the growth temperature for clone M×N. In contrast, no acclimation of *R*_*d*_ was observed for clone M×B. The contrasting thermal acclimation capacity observed for the two hybrid poplar clones may be associated to the modulation capacity of the density of mitochondria and the expression of the alternative oxidative pathway (AOX) [49, 53]. In fact, the increase of growth temperature generally induces a decrease in AOX protein abundance and density of mitochondria [3].

Thermal acclimation of non-photorespiratory mitochondrial respiration in the light (*R*_*day*_) is an important and less investigated component of thermal acclimation of *A*_*n*_ [6]. *R*_*day*_ is generally strongly correlated with *R*_*d*_ and assumed to be half *R*_*d*_ [6, 54]. The temperature response of *R*_*day*_ and its acclimation to growth temperature has been found to mirror those of *R*_*d*_ for some species [6]. Therefore, we may expect the involvement of *R*_*day*_ in the observed acclimation of *A*_*n*_ of our study if we assume a similar response of *R*_*day*_ compared to *R*_*d*_.

### Thermal response of photosynthetic biochemical limitations

The effect of growth temperature on temperature response curve of apparent *V*_cmax_ and *J* in terms of their values at reference temperature of 25°C, their *T*_*opt*_ and their activation energy is species-dependent as reported by recent studies [6, 9, 19, 23, 55, 56]. In our study, the apparent *V*_cmax_^25^ stimulated by warm growth temperature for clone M×B, might explain the noticeable increase of *A*_*n-opt*_ (up to 50%) by warmer growth conditions under high N level. In parallel, the small decrease in the apparent *V*_cmax_^25^ at warm growth conditions observed for clone M×N might explain the observed similar *A*_*n-opt*_ under the two growth temperatures. These results are in agreement with the findings of other studies showing a similar or a greater *V*_cmax_^25^ when growth temperature increased [6, 33, 56, 57]. In contrast, the apparent *J*_max_^25^ decreased at warmer growth temperature as reported for *Populus balsamifera* [33] and other tree species [6, 56, 58].

Hikosaka et al.s [9] suggested an increase in the activation energy of *V*_*cmax*_ (*Ea*) with an increase in growth temperature as an explanatory mechanism of thermal acclimation of *A*_*n*_ (at least by the increase of *T*_*opt*_ with growth temperature). Our results are diverging with this postulate since we observed no change in *E*_*a*_ for clone M×N and a remarkable decrease of *E*_*a*_ for clone M×B. However, the patterns we observed have been reported for several species including *Populus tremuloides* [34], *Populus balsamifera* [33] and *Corymbia calophylla* [57].

The temperature optimum (*T*_*opt*_) of apparent *V*_cmax_ and *J* acclimated to growth temperature (Fig 4) as observed for other species [17, 23] and may have contributed in the observed acclimation of *A*_n_ (Fig 2).

The adjustment of leaf nitrogen invested in soluble *vs*. insoluble proteins in response to change in growth temperature, inferred from *J*_max_^25^ to *V*_cmax_^25^ ratio, can be achieved through the maintenance of an optimal balance between the rate of photosynthetic carboxylation *vs*. RuBP regeneration. This mechanism allows plants to maximize the photosynthetic rate at a given growth temperature [23, 55]. Therefore, the decrease of *J*_max_^25^:*V*_cmax_^25^ ratio consequent to an increase of growth temperature has been reported to significantly contribute to thermal acclimation of *A*_*n*_ [17, 23, 48, 56]. In our study, this pattern occurred for clone M×B under high N level which increased both *V*_cmax_^25^ and *A*_*n-opt*_. Conversely, the lack of modulation of *J*_max_^25^:*V*_cmax_^25^ ratio for clone M×N may have contributed to the observed decrease in *V*_cmax_^25^ and to the maintenance of *A*_*n-opt*_. Under low N level, *A*_*n-opt*_ of M×B increased under warmer conditions without any change of the *J*_max_^25^:*V*_cmax_^25^ ratio. Therefore, the increase of *V*_cmax_^25^ and *A*_*n-opt*_ under the warm growth temperature cannot be attributed only to the shift in *J*_max_^25^:*V*_*c*max_^25^ ratio.

### Stomatal conductance

The contribution of diffusional limitations to thermal acclimation of *A*_n_ remains non-well quantified for several species, including *Populus*. Our results demonstrate that the modulation of *g*_s_ (the shape of the relationship between *g*_s_ and *T*_*leaf*_ and the value of *g*_*s*_ at growth temperature) in response to changes in growth temperature (Fig 5) may contribute to the observed thermal acclimation of *A*_n_ as previously reported [33, 56, 57]. Also, our results suggest that the stomatal acclimation to growth temperature may be clone-specific and may have a significant impact on clone response to warming depending on soil water status. The CO_2_ diffusion in the mesophyll shares the same pathways of water transport from mesophyll to the atmosphere [38, 59] and may lead to a similar response of stomatal and mesophyll conductance to growth conditions. Moreover, a link between mesophyll conductance (*g*_*m*_) and hydraulic conductance has been reported as well [54, 59], suggesting that the observed response of *g*_s_ to growth temperature may have originated from modulation of *g*_m_ and hydraulic functioning.

### RuBisCO and RuBisCO activase amounts in response to experimental warming

The RuBisCO content in our study was quite sensitive to nitrogen level but not to growth temperature. Neither thermal acclimation of *A*_*n*_ (*T*_*opt*_ and *A*_*n-opt*_) nor *J*_max_^25^:*V*_cmax_^25^ ratio was affected by RuBisCO content. The absence of any effect of RubisCO content on traits related to thermal acclimation of *A*_*n*_ has also been reported by Weston et al.’s [24] and Kruse et al.’s [60], while other studies found a significant decrease in *V*_*cmax*_^*25*^ linked to a decrease in RuBisCO and leaf nitrogen content [16, 17]. Thus, the relationship between the change in RuBisCO content in response to growth temperature and thermal acclimation of *A*_*n*_ via the modulation of photosynthetic capacity attributes (*V*_*cmax*_^*25*^ and *J*_*max*_^*25*^) is, most likely, depending on species and environmental parameters (e.g. nitrogen availability). Indeed, CO_2_ conductance, *R*_*day*_, the variation of RuBisCO activase content and the temperature dependency of RuBisCO kinetic properties have been reported to be determinant factors of the *V*_cmax_^25^ response to growth temperature and consequently thermal acclimation of *A*_*n*_[6, 20, 45, 61]. The increase of leaf RuBisCO activase amount by increased growth temperature has been reported for several tree species[24, 27–31]. In our study, the hypothesized increase of RARCA at warmer growth temperature was observed only for clone M×N at high N and for clone M×B at low N. Likely, having more than three replicates per treatment (n = 3) could make this trend more obvious. Besides, our results demonstrated that the increase of *RARCA* under warm conditions resulted mainly from increased synthesis of the short isoform suggesting that the two isoforms operate at different temperature optima.

Overall, we think that using a larger set of clones would help assess the adaptive value of thermal acclimation and dissect its genetic control and molecular mechanisms [62]. Also, our study highlighted the complexity of assessing thermal acclimation of photosynthesis as a multi-trait process and the need for further investigation regarding the involvement of mesophyll conductance and hydraulic conductivity as well as the expression of the photosynthesis-related pool of proteins to better understand the mechanistic basis of the observed trends.

In conclusion, the observed thermal acclimation of photosynthesis under our experimental conditions was clearly related to the modulation of photosynthetic capacity and *g*_*s*_ in response to growth temperature. The modulation of the photosynthetic capacity was mainly linked to RuBisCO activase but not RuBisCO content. On the other hand, our results do not support the involvement of leaf N status in thermal acclimation of *A*_*n*_ and *R*_*d*_.

## Supporting information

S1 Fig Western blot of RuBisCO and RuBisCO activase for two hybrid poplar clones (M×N and M×B) under combinations of growth temperature (23°C and 33°C) and nitrogen level (high level: HN and low level: LN). (PDF)

S1 File ACi curve data. (TXT)

S1 Table Analysis of variance, *F* and *P* values for thermal acclimation-related traits. (PDF)

S2 Table *P* value and R^2^ of curves in Figs 1, 2 and 3. (PDF)
