# Supplementary material for: Thermal acclimation of photosynthetic activity and RuBisCO content in two hybrid poplar clones
Source: PLoS One. 2019 Feb 11;14(2):e0206021. doi: 10.1371/journal.pone.0206021 (PMC6370183; doi:10.1371/journal.pone.0206021)
Supplement: S1 Fig — (PDF) [file pone.0206021.s001.pdf]

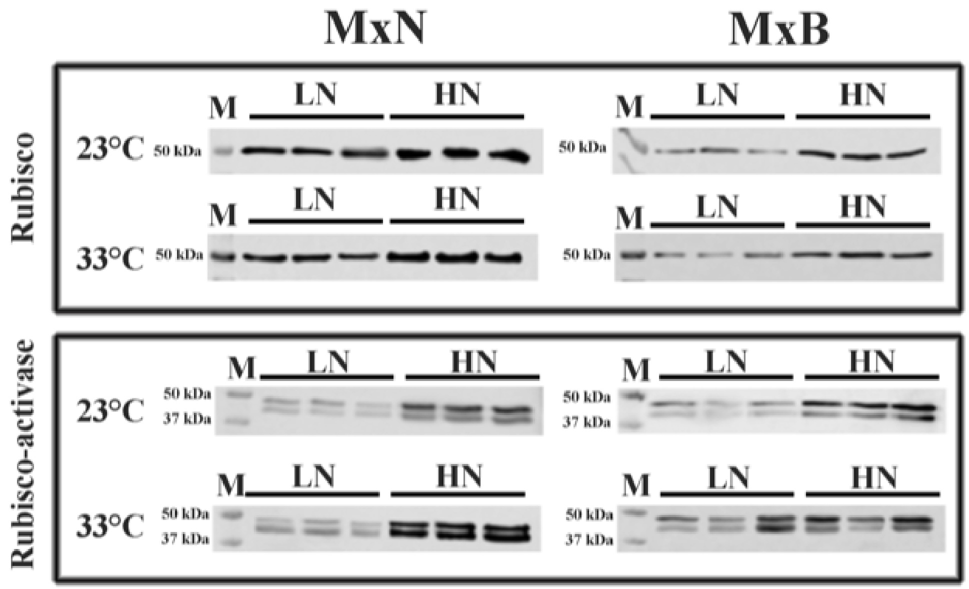

S1 Fig. Western blot of RuBisCO and RuBisCO activase for two hybrid poplar clones (M×N and M×B) under combinations of growth temperature (23°C and 33°C) and nitrogen level (high level: HN and low level: LN). 20 µg of total soluble proteins extracted from leaves were loaded per well. Immunoblots were probed with anti RuBisCO or anti RuBisCO activase antibodies along with conjugated secondary antibodies. The second sample for clone. M×B at H33 (well 5) was excluded in the statistical analysis (Hat value).
