# Supplementary material for: Thermal acclimation of photosynthetic activity and RuBisCO content in two hybrid poplar clones
Source: PLoS One. 2019 Feb 11;14(2):e0206021. doi: 10.1371/journal.pone.0206021 (PMC6370183; doi:10.1371/journal.pone.0206021)
Supplement: S1 Table — (PDF) [file pone.0206021.s003.pdf]

S1 Table. Analysis of variance, *F* and *P* values for thermal acclimation-related traits.

		A_{n_opt}		T_{opt}		A_{n_growth}		g_{s_growth}		$J_{max}^{25}:V_{cmax}^{25}$		N_{area}	
Source	DF	*F*	*P Value*	*F*	*P Value*	*F*	*P Value*	*F*	*P Value*	*F*	*P Value*	*F*	*P Value*
T	1	8.2	0.016	44.31	<.001	2.27	0.162	1.15	0.285	4.63	0.063	2.38	0.146
N	1	31.77	**0.011**	1.28	0.340	18.17	0.023	0.67	0.474	7.21	0.074	49.29	0.005
T*N	1	0.16	0.697	4.53	0.059	0	0.997	0.66	0.417	4.39	0.069	2.35	0.149
Clone	1	14.21	0.003	5.41	0.042	8.47	0.015	23.12	<.001	11.51	0.009	17.73	0.001
Clone*T	1	8.46	0.015	0.32	0.582	9.63	0.011	27.56	<.001	0.8	0.398	3.67	0.077
Clone*N	1	0.01	0.932	0.68	0.427	0.05	0.821	1.92	0.168	3.96	0.081	0	0.991
Clone*T*N	1	4.03	0.072	0.24	0.632	1.83	0.205	10.11	0.001	0.13	0.727	2.34	0.150

T, growth temperature; N, soil N. For abbreviation, see table 1.
