# Supplementary material for: Thermal acclimation of photosynthetic activity and RuBisCO content in two hybrid poplar clones
Source: PLoS One. 2019 Feb 11;14(2):e0206021. doi: 10.1371/journal.pone.0206021 (PMC6370183; doi:10.1371/journal.pone.0206021)
Supplement: S2 Table — (PDF) [file pone.0206021.s004.pdf]

S2 Table. *P* value and R^2 of curves in figures 1, 2 and 3.

		A_n		R_d		g_s		V_{cmax}		J	
		P value	R^2	*P* value	R^2	*P* value	R^2	*P* value	R^2	*P* value	R^2
**Clone**	**Treatment**										
M×B	H23	0.011	0.84	0.001	0.96	0.001	0.89	0.039	0.88	0.035	0.94
	L23	0.010	0.95	0.001	0.95	0.003	0.87				
	H33	0.010	0.88	0.001	0.96	0.01	0.85	0.001	0.98	0.001	0.98
	L33	0.084	0.81	0.001	0.97	0.07	0.95	0.001	0.98	0.001	0.95
M×N	H23	0.001	0.96	0.001	0.98	0.02	0.95	0.015	0.95	0.001	0.98
	L23	0.021	0.89	0.001	0.98	0.01	0.91				
	H33	0.015	0.92	0.001	0.98	0.02	0.91	0.001	0.98	0.001	0.97
	L33	0.004	0.92	0.001	0.98	0.007	0.93	0.001	0.95	0.001	0.96

A_n, R_d and g_s response to leaf temperature were fitted using a polynomial equation of order 2 (quadratic) while V_{cmax} and J were fitted using the Arrhenius formula, following equations 7 or 8 depending on the presence or not of deactivation at high temperatures.
